# 

**Published:** 1984-04

**Authors:** 


					MEDICO
CHIRURGICAL
APRIL 1984

				

